# Isoespintanol Isolated from *Oxandra* cf. *xylopioides* (Annonaceae) Leaves Ameliorates Pancreatic Dysfunction and Improves Insulin Sensitivity in Murine Model of Fructose-Induced Prediabetes

**DOI:** 10.3390/plants14050745

**Published:** 2025-03-01

**Authors:** Sherley Catherine Farromeque Vásquez, Luisa González Arbeláez, Benjamín Rojano, Guillermo Schinella, Bárbara Maiztegui, Flavio Francini

**Affiliations:** 1 CENEXA (Centre for Experimental and Applied Endocrinology—UNLP CONICET CCT La Plata—CEAS CICPBA), School of Medicine, Street 60 and 120, La Plata 1900, Argentina; sfarromeque@med.unlp.edu.ar (S.C.F.V.); bmaiztegui@cenexa.org (B.M.); 2 CIC (Centre for Cardiovascular Research—UNLP CONICET CCT La Plata), School of Medicine, Street 60 and 120, La Plata 1900, Argentina; luisafarbelaez@med.unlp.edu.ar; 3 Universidad Nacional de Colombia, Sede Medellín, Facultad de Ciencias, Laboratorio de Ciencia de Alimentos, Medellín 050012, Colombia; brojano@unal.edu.co; 4 UNLP—School of Medicine, Cathedra Basic Pharmacology, Street 60 and 120, La Plata 1900, Argentina; schinell@med.unlp.edu.ar; 5 UNAJ-CICPBA, Institute of Health Sciences, Av. Calchaquí 6200, Florencio Varela 1888, Argentina

**Keywords:** prediabetes, *Oxandra* cf. *xylopioides*, pancreatic islets, isoespintanol

## Abstract

In rats, a fructose-rich diet triggers endocrine-metabolic disturbances similar to those present in human prediabetes. We evaluated the protective effect of isoespintanol, a monoterpene isolated from *Oxandra* cf. *xylopioides* (Annonaceae), on pancreatic islet. Rats were kept for three weeks with a standard commercial diet and tap water (C), plus 10% fructose (F), or F plus isoespintanol (I; 10 mg/kg, *i.p*.). Glycemia, triglyceridemia, total cholesterol, HDL-cholesterol, insulin resistance index (IRX), and glucose tolerance tests were determined. Glucose-stimulated insulin secretion (GSIS) and gene expression of insulin signalling mediators (insulin receptor -*IR-, IRS1/2, PI3K*), oxidative stress (*SOD-2, GPx, GSR*, 3’-nitrotyrosine), inflammation (*TNF-α, IL-1β, PAI-1*), mitochondrial function (*Bcl-2, mtTFA, PGC-1α*), and apoptosis markers were evaluated in pancreatic islets. The F group increased triglyceridemia, non-HDL-cholesterol, and IRX, and decreased HDL-cholesterol and impaired glucose tolerance, with alterations reversed by isoespintanol administration (*p* < 0.05). Isoespintanol normalized higher GSIS recorded in the F group. F decreased mRNA levels of insulin signalling mediators and mitochondrial function markers, and increased the expression of inflammatory, apoptotic, and oxidative stress markers, alterations that were significantly reversed by isoespintanol. Current results suggest that isoespintanol improved insular oxidative stress and inflammation by affecting the IR-PI3K pathway, which plays a pivotal role in insulin resistance development, underlying its therapeutic potential for the prevention of type 2 diabetes before its onset (prediabetes).

## 1. Introduction

A global diabetes epidemic poses a significant public health challenge. According to the International Diabetes Federation [1], an estimated 463 million adults worldwide lived with diabetes in 2019, with projections reaching 700 million by 2045 if preventative measures are not implemented. Notably, type 2 diabetes (T2D) accounts for 90% of all cases [1]. The pathophysiology of T2D involves a complex interplay of genetic, environmental, and lifestyle factors leading to insulin resistance and pancreatic beta-cell dysfunction, ultimately resulting in chronic hyperglycaemia and serious potential complications [2]. Fortunately, prediabetes, a metabolic precursor to T2D identified by fasting blood glucose levels, glucose tolerance tests, or glycosylated hemoglobin (HbA1c) levels [3], offers an earlier window for intervention. Diagnosis and appropriate management of prediabetes can significantly delay or prevent T2D progression [4].

Lifestyle modifications, particularly promoting healthy diet and regular physical activity, have proven effective in managing prediabetes [4]. However, trends like increased fructose consumption in industrialized societies are fuelling concerns. The widespread use of high-fructose corn syrup (HFCS) in processed foods and beverages has demonstrably contributed to this rise [5]. Several studies have linked fructose-rich diets to the growing epidemic of diabetes, obesity, and other metabolic disorders [6,7,8].

In previous research by our laboratory, we employed a murine model (rats) to demonstrate that administering fructose for 3 weeks induced dyslipidemia, impaired glucose tolerance, insulin resistance, liver steatosis, and pancreatic β-cell dysfunction, mirroring the abnormalities observed in human metabolic syndrome [9,10].

While synthetic medications can effectively manage diabetes, they may come with undesirable side effects. This has spurred renewed interest in exploring natural plant products as potential sources for new and cost-effective therapies [11,12]. The search for new compounds with defined structural characteristics and biological properties is a permanent challenge for professionally studying natural products. In this sense, several phytochemicals with anti-diabetic properties within medicinal plants, encompassing major groups like alkaloids, flavonoids, terpenes, and more [13,14,15,16], have been identified. However, clinical data regarding the efficacy of these compounds on prediabetes remains inconsistent. The present study focuses on isoespintanol (2-Isopropyl-3,6-dimethoxy-5-methylphenol), a monoterpene isolated from the leaves of *Oxandra cf xylopioides*. Isoespintanol is a natural compound with demonstrated pharmacological activities, including anti-inflammatory [17], antioxidant [18], antispasmodic [19], and cardioprotective [20] effects. Previous studies demonstrated that this monoterpene acts pharmacologically through different pathways. However, the possible effects on pancreatic islets, a main player in glycemic control and in turns in T2D, remain unknown. 

Our previous research demonstrated that isoespintanol administration in fructose-fed rats normalized fructose-induced hepatic metabolic dysfunction, oxidative stress, and inflammation [18]. Given the established effects of fructose in the induction of a pathological triad, inflammation, insulin resistance and oxidative stress, and considering that isoespintanol possess anti-inflammatory and antioxidant activities, our hypothesis is that isoespintanol, acting of pancreatic islets of Langerhans, a main player in glycemic control, could disrupt the above-mentioned triad thus protecting from the deleterious of a fructose overload. Therefore, this study aims to evaluate the potential protective effects of isoespintanol on pancreatic islets of prediabetic rats fed a rich fructose diet for three weeks. 

## 2. Results

### 2.1. Body Weight, Water and Food Intake, Calories Consumed

Rats treated with 10% fructose (F and I groups) drank a significantly higher volume than control rats after 21 days of treatment. In contrast, the C rats ate a higher amount of solid food compared to fructose-treated rats (Table 1). However, their caloric intakes were comparable without significant differences (Table 1). Although there were no significant differences in body weight gain between the C and the F groups, isoespintanol treated animals showed a decrease in body weight gain compared to the F rats (Table 1).

### 2.2. Serum Parameters

Although serum glucose levels were similar among the groups, the F rats exhibited a significant increase in triglyceride and non-HDL-cholesterol levels and the IRX related to the C animals (*p* < 0.05) (Table 2). These changes, together with the significant decrease in HDL-cholesterol levels, evidence a dyslipidemic and insulin resistance state in the F group. These endocrine-metabolic alterations induced by fructose were significantly reversed with the administration of isoespintanol (Table 2).

### 2.3. Intraperitoneal Glucose Tolerance Test (IGTT)

Glucose concentrations, measured at 0, 15, 30, 60, and 120 min following the glucose challenge in the IGTT assay, were used to calculate the area under the curve (AUC). Results showed that the AUC was significantly higher in the F group compared to the C rats (Table 1; *p* < 0.05). The impaired glucose tolerance induced by fructose was significantly restored with isoespintanol treatment (Table 1; *p* < 0.05).

### 2.4. Pancreatic Islet Determinations

#### 2.4.1. Glucose-Induced Insulin Secretion

**Figure 1:**
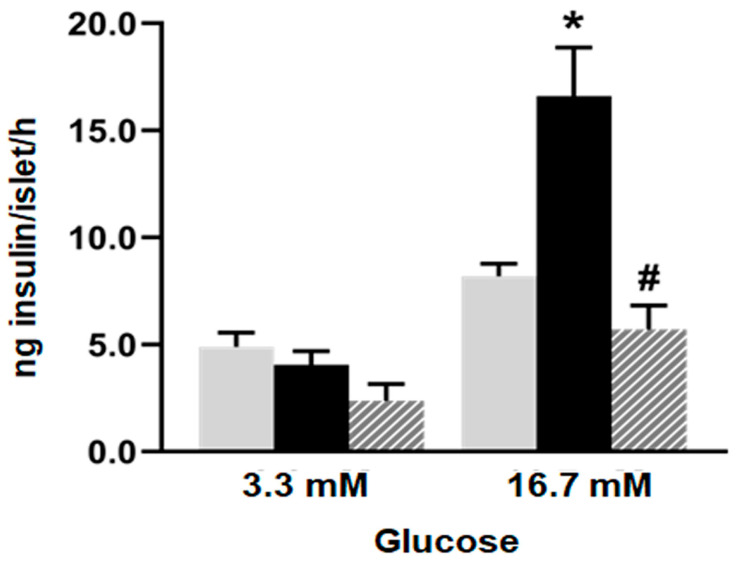
Glucose-induced insulin secretion. Insulin secretion in response to 3.3 and 16.7 mM glucose by islets isolated from C (grey bars), F (black bars), and I (lined bars) rats. Data are shown as means ± SEM from three independent experiments. * *p* < 0.05 vs. C; # *p* < 0.05 vs. F. Insulin released into incubation media was expressed as ng of insulin per islet/h.

No differences in insulin secretion were registered among the experimental groups when glucose 3.3 mM was assayed (basal condition). However, islets from the F group released higher amounts of insulin compared to the C group when they were incubated at a stimulus glucose concentration of 16.7 mM (Figure 1; *p* < 0.05). Notably, the administration of isoespintanol to the F rats significantly decreased insulin secretion, reaching values like those of the C animals.

#### 2.4.2. Gene and Protein Expression of Insulin Pathway Mediators

**Figure 2:**
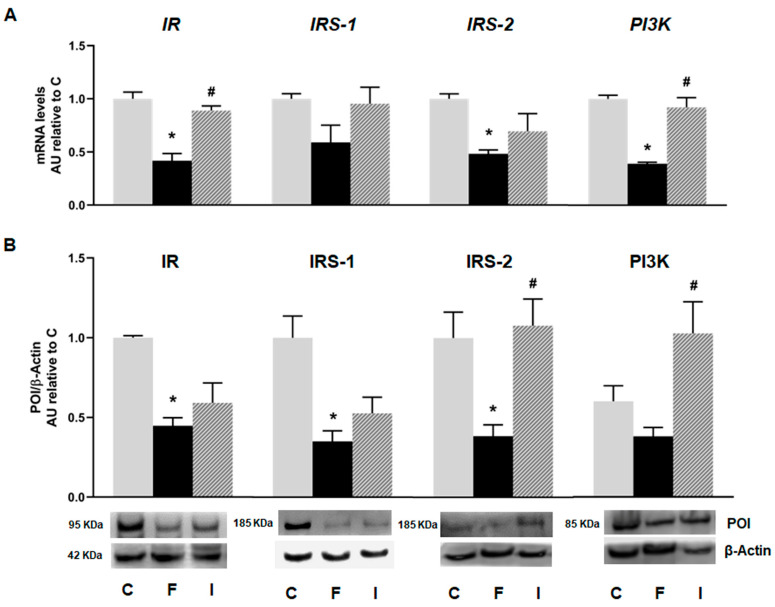
Gene expression of *IR, IRS-1, IRS-2,* and *PI3K* evaluated in pancreatic islets isolated from C (grey bars), F (black bars), and I (lined bars) rats. (**A**) mRNA relative expression measured by RT qPCR. β-actin employed as housekeeping gene. Data are shown as arbitrary units (AUs) with respect to mRNA level measured in C group. Bars represent means ± SEM from three independent experiments. (**B**) Protein levels determined by Western blot in islet homogenates from all experimental groups. Representative blot is shown in each case. Bars represent means ± SEM expressed in arbitrary units (AUs) as ratio between protein of interest (POI) and β-actin band intensity. * *p* < 0.05 vs. C; # *p* < 0.05 vs. F. *n* = 6 rats/group.

Islets isolated from the F animals, evinced a decrease in the mRNA levels of insulin cascade mediators, including insulin receptor (*IR*), its substrate (*IRS-2*), and the downstream mediator *PI3K* compared to the C group (*p* < 0.05; Figure 2A). Even when *IRS-1* showed a decrease in mRNA level, it did not attain statistical significance. Accordingly, protein analysis revealed that the F rats also evinced a significant decrease in IR, IRS-1, and IRS-2 protein levels (*p* < 0.05; Figure 2B). These alterations induced by fructose were almost completely reversed with isoespintanol administration. Notably, the protein levels of IRS-2 and PI3K were significantly increased in the I rats compared to the F ones (Figure 2B).

#### 2.4.3. Inflammation Markers

**Figure 3:**
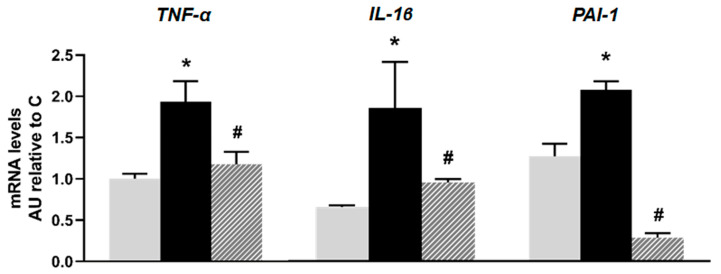
mRNA relative expression of *TNF-α, IL-1β,* and *PAI-1* measured by RT- qPCR in pancreatic islets isolated from C (grey bars), F (black bars), and I (lined bars) rats. β-actin was used as internal standard. Values were expressed in arbitrary units (AUs) with respect to mRNA level determined in C group. Bars represent means ± SEM from three independent experiments. * *p* < 0.05 vs. C; # *p* < 0.05 vs. F. *n* = 6 rats/group.

Regarding inflammation markers, islets isolated from the F animals showed a significant increase in mRNA levels of *TNF-α, IL-1β,* and *PAI-1* compared to the C group (*p* < 0.05). The inflammatory profile induced by fructose was significantly reversed with isoespintanol treatment (Figure 3).

#### 2.4.4. Oxidative Stress Markers

**Figure 4:**
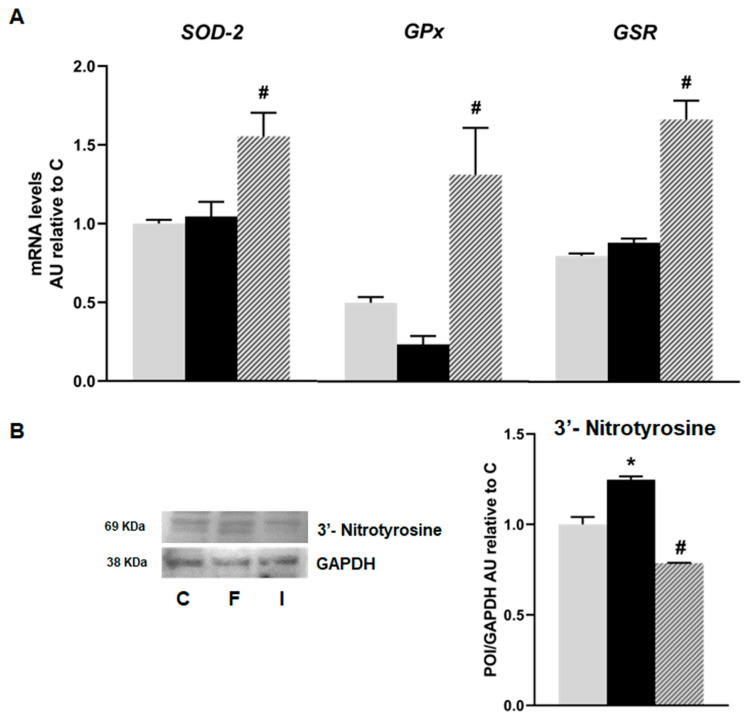
Gene expression of *SOD-2, GPx,* and *GSR* evaluated in pancreatic islets isolated from C (grey bars), F (black bars), and I (lined bars) rats. (**A**) mRNA relative expression measured by RT qPCR. β-actin was used as internal standard. Values were expressed in arbitrary units (AUs) with respect to mRNA level measured in C group. Bars represent means ± SEM from three independent experiments. (B) Protein levels of 3’- Nitrotyrosine determined by Western blot. Data are shown as means ± SEM in arbitrary units (AU) as the ratio between the protein of interest (POI) and GAPDH band intensity. * *p* < 0.05 vs. C; # *p* < 0.05 vs. F. *n* = 6 rats/group.

mRNA levels of oxidative stress markers such as Superoxide dismutase 2 (*SOD-2*), Glutathione peroxidase (*GPx*), and Glutathione reductase (*GSR*) showed no significant differences between the C and F groups. However, islets from the I animals exhibited a significant increase in mRNA levels, compared to the F group (*p* < 0.05; Figure 4A). Additionally, a significant increase in immunoreactive 3′ nitrotyrosine (a peroxynitrite production and protein nitration marker) protein was recorded in the F rats compared to the C group. This effect was completely reversed by isoespintanol administration, evidencing a significant decrease in oxidative stress (Figure 4B).

#### 2.4.5. Mitochondrial Function Markers

**Figure 5:**
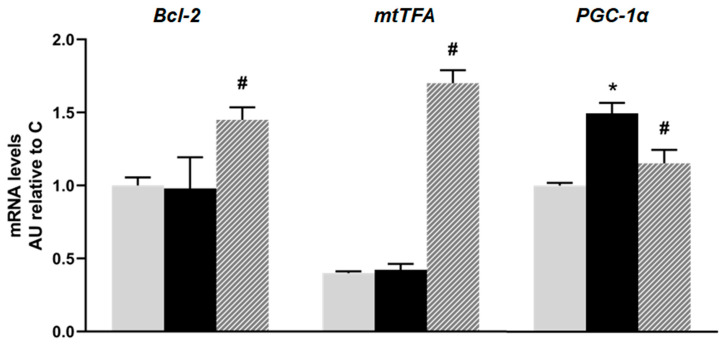
mRNA relative expression of *Bcl-2, mtTFA,* and *PGC-1α,* measured by RT- qPCR in pancreatic islets isolated from C (grey bars), F (black bars), and I (lined bars) rats. β-actin was used as internal standard. Values were expressed in arbitrary units (AUs) with respect to mRNA level determined in C group. Bars represent means ± SEM from three independent experiments. * *p* < 0.05 vs. C; # *p* < 0.05 vs. F. *n* = 6 rats/group.

No significant differences were observed in the mRNA levels of *Bcl-2* and mitochondrial transcription factor A (*mtTFA*) between the C and F animals; however, they were significantly increased in the rats treated with isoespintanol. On the other hand, the mRNA levels of peroxisome proliferator-activated receptor gamma 1-alpha coactivator (*PGC-1α*) were higher in the F rats compared to the C ones, and there was a significant decrease in *PGC-1α* gene expression in the I animals compared to the F group (*p* < 0.05; Figure 5).

#### 2.4.6. Apoptosis Markers

**Figure 6:**
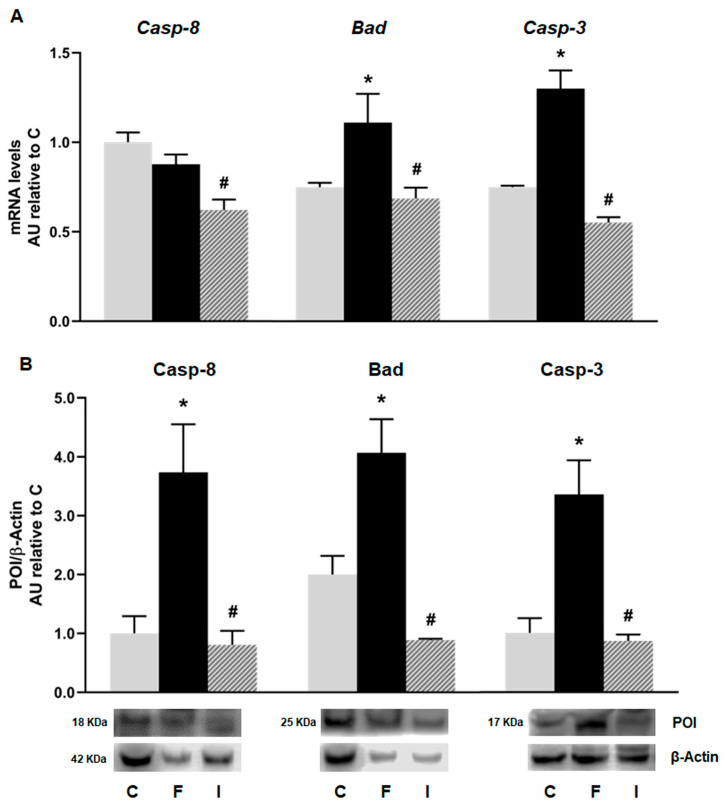
Gene expression of Caspase-8 (*Casp-8*), *Bad*, and Caspase-3 (*Casp-3*) evaluated in pancreatic islets isolated from C (grey bars), F (black bars), and I (lined bars) rats. (**A**) mRNA relative expression measured by RT qPCR. *β-actin* was used as housekeeping gene. Values were expressed in arbitrary units (AUs) with respect to mRNA level determined in C group. Data are shown as means ± SEM from three independent experiments. (**B**) Protein levels determined by Western blot in islet homogenates from all experimental groups. Representative blot is shown in each case. Bars represent means ± SEM expressed in arbitrary units (AUs) as ratio between protein of interest (POI) and β-actin band intensity. * *p* < 0.05 vs. C; # *p* < 0.05 vs. F. *n* = 6 rats/group.

Although the mRNA levels of *Caspase 8* (the initiator apoptotic marker of the extrinsic apoptosis pathway) showed no significant difference between the C and F rats, when analyzing their protein levels, the F animals showed that they were significantly increased compared to the C group. Accordingly, both mRNA and protein levels of Bad and the active (cleaved) form of Caspase 3 (the effector protein of apoptotic pathway) were significantly increased in the F rats compared to the C animals. The administration of isoespintanol was able to reverse the apoptotic effect induced by fructose, reducing the gene expression (mRNA and protein levels) of all the apoptosis markers studied (Figure 6).

## 3. Discussion

Our data showed, for the first time, that the monoterpene isoespintanol isolated from *Oxandra* cf. *xylopioides* administered to prediabetic rats, normalized pancreatic islet’s dysfunction and insulin resistance triggered by a fructose-rich diet. The current study also suggests that this compound improved insular oxidative stress and inflammation by affecting the IR-PI3K signalling pathway, which plays a pivotal role in insulin resistance development.

Several monoterpenes and their derivates were proved to have potential anti-diabetic effects acting on different pathways in pancreatic β-cells [21]. In STZ-diabetic rats, β-caryophyllene exerts an anti-diabetic effect through the induction of insulin secretion in pancreatic β-cells [22] and the same model demonstrated an improvement of β-cell function and alleviation of insulin resistance, probably decreasing oxidative stress in different tissues, including the pancreas [23]. In our model, isoespintanol-treated rats had improved insulin resistance and normalized in vitro insulin secretion, probably involving the IR-PI3K-AKT pathway. In addition, Aguilar-Ávila et al. showed that β-caryophyllene improved insulin secretion in diabetic mice through the amelioration of inflammation and oxidative stress in β-cells [24], a similar phenomenon recorded in our rats administrated with isoespintanol. On the other hand, it was demonstrated that the monoterpenoid geraniol, found in several essential oils, improves β-cell function and insulin secretion in diabetic rats together with an intracellular increase in NADPH content, an important player in the generation of the intracellular antioxidant Glutathione [25]. In this sense, being the pancreatic islet micro-organs with a poor antioxidant defence, and therefore vulnerable to oxidative stress, antioxidant drugs were reported to protect them from the deleterious effects of hyperglycemia [26]. In effect, it was described that high glucose concentrations increased intracellular peroxide levels in human islets and in the pancreatic β-cell line HIT-T15, features that were blunt by the adenoviral overexpression of *GPx* [27]. The protective effect of GPx is ascribed to its ability to catabolize both H_2_O_2_ and lipid peroxides. The level of this antioxidant in β-cells is extremely low and its overexpression enhanced protection against oxidative stress [28].

Additionally, reducing sugars, such as fructose, are known to play a role in the initiation of apoptosis in pancreatic β-cells. Susuki et al. demonstrated that fructose increases H_2_O_2_ levels and lipid peroxidation in hamster islet tumour (HIT) cells because of the inactivation of the activity or expression of GPx [29]. The current results showed lower *GPx* mRNA levels in islets from the fructose-treated rats compared to islets from the control rats (Figure 4A). Isoespintanol treatment significantly increased *GPx* expression in islet cells, demonstrating its potential to counteract the negative effects of fructose on *GR* expression in islet cells (Figure 4A).

On the other hand, elevating GR activity is a common cellular strategy to combat oxidative stress, since GR regenerates GSH from GSSG [30]. The capacity to replenish GSH was shown to be more critical for cell viability than the steady-state GSH level [31]. In this sense, we found that isoespintanol treatment significantly increased GR mRNA expression in islet cells.

Our data show that isoespintanol exerts anti-nitrosative actions (Figure 4B). This effect could be related, as we demonstrated previously, to the fact that this compound can change the NO/ONOO− balance toward NO [20] and/or increasing the expression of *GPx* and *GR*.

Complementary, Prasad found in STZ-diabetic rats that the geraniol improved diabetic neuropathy probably due to their anti-inflammatory and antioxidant effects [32]. In this sense, it has was shown that α-Humulene improved β-cell function and survival in diabetic rats, probably through its antioxidant properties [33]. Taken together, these results suggest that the protective effects of isoespintanol on insulin signalling and secretion recorded in our experiments could be due to an improvement in the oxidative and inflammatory state of pancreatic islets.

Other monoterpenes, like α-pinene and linalool were shown to reduce glycemia in alloxan-induced diabetic mice [34] or glucose uptake in STZ-induced diabetic rats [35] probably also due to their antioxidant and anti-inflammatory actions. Additionally, limonene, another terpene that mitigates insulin resistance and diabetes progression, was found to ameliorate insulin resistance in an HFD model by a mechanism that involved reduction in triglycerides and free fatty acid deposition that in turn normalized β-cell mass and insulin secretion [36]. In our case, even when prediabetic rats are normoglycemic, isoespintanol alleviates insulin resistance and glucose intolerance together with a normalization of plasmatic TG levels, thus suggesting a similar mechanism. On the other hand, using STZ-induced diabetic rats, Madhuri and Naik demonstrated that the antihyperglycemic and antihyperlipidemic activities of borneol are probably mediated by its positive effect on insulin secretion by blocking the ATP-sensitive K+ channels and increasing intracellular Ca^2+^ [37]. Thus, the improvement recorded in the circulating TG levels in our isoespintanol treated rats could also be related to the secretagogue effect of the mentioned compound.

On the other hand, in recent years, it has been suggested that mitochondria have a pathogenic role in metabolic abnormalities such as obesity, metabolic syndrome, and T2D. Several publications have shown that mitochondrial dysfunction contributes to inflammation and oxidative stress in metabolic syndrome [38,39]. In this sense, the monoterpene linalool was demonstrated as a potential protective compound in oxidative stress conditions by preserving mitochondrial membrane potential and uncoupling respiration [40]. This is in line with our current results in which isoespintanol increased *mtTFA* and *Bcl-2* expression, thus suggesting that in our model, this compound also protects mitochondrial function.

In conclusion, our results demonstrated for the first time that the administration of the monoterpene isoespintanol to prediabetic rats prevented pancreatic islet dysfunction induced by an unhealthy diet (rich in fructose). The antioxidant and anti-inflammatory effects of this compound could improve the insulin resistance phenotype, probably acting at the IR-PI3K signalling pathway, thus disrupting the vicious circle of insulin resistance, oxidative stress, and inflammation, at least in our rat model. On the other hand, as described for other monoterpenes, isoespintanol could improve mitochondrial function. In consequence, even when the topic merits further research to validate these findings in human subjects, isoespintanol arises as a promising candidate to develop new therapeutic tools to treat prediabetes.

## 4. Materials and Methods

### 4.1. Chemicals and Drugs

Collagenase-P, FastStart SYBR Green Master mix, and Bovine Serum Albumin (fraction V) were obtained from Roche Diagnostics GmbH (Mannheim, Germany). Primary antibodies were supplied by Santa Cruz Biotechnology, Inc. (Santa Cruz, CA, USA), Cayman Chemical (Michigan, MI, USA), Sigma (St. Louis, MA, USA) and Merck Millipore (Burlington, MA, USA).

### 4.2. Plant Material

The material studied from the plant *Oxandra cf xylopioides*, belonging to the Annonaceae family, was collected in Monteria, Cordoba, Colombia, and identified by Prof. Francisco Javier Roldan Palacio, University of Antioquia. A voucher specimen was deposited at the Botanical Garden Joaquin Antonio Uribe, Medellin, Colombia (voucher number 037852).

Isoespintanol was obtained from the leaves of *Oxandra cf. xylopioides*, as previously described [17], after extraction with petroleum ether by percolation and dried by rotary evaporation. The extract was subjected to chromatographic columns by gravity, eluting with hexane–dichloromethane mixtures and, finally, recrystallizing [16]. The purity of the sample was assessed by HPLC analyses, for which isoespintanol was dissolved in 10.0 mL of ethanol (at a final concentration of 500 mg/L) and purity quantified by HPLC-DAD (Shimadzu Prominence^®^, Tokyo, Japan). Isocratic elution was conducted with methanol/water (60:40); flow rate of 1.0 mL/min; with a LiChrospher^®^ 100 RP-18 column (Merck, Darmstadt, Germany) as stationary phase at 35 °C; and an injection volume of 20 µL. Retention time for isoespintanol was 9.28 min and >99% purity.

### 4.3. Experimental Groups

Two months old male Sprague Dawley rats were kept in a controlled environment (23 ± 1 °C, 50% humidity, and fixed 12 h light/dark cycle). Thirty rats (250–300 g) were randomly divided into three experimental groups: control (C) with free access to a standard commercial diet and tap water; fructose-rich diet (F) with standard commercial diet supplemented with 10% (*w/v*) fructose in the drinking water for three weeks; and the isoespintanol group (I), fed with the same diet as the F group, plus a daily intraperitoneal injection of isoespintanol (10 mg/kg/day in DMSO, final volume 200 µL, i.p) during the last 5 days of treatment, as described [18]. Previous trials confirmed the safety of DMSO as a vehicle in animals, as well as the dose employed and the duration of treatment. During the 21-day treatment period, the body weights and water and food consumption of all experimental groups were recorded. The protocols used for the animal experiments are in accordance with the ethical principles and guidelines for experimental animals from Swiss Academy of Medical Science. They were evaluated and approved by the Animal Welfare Committee (CICUAL) of La Plata School of Medicine, National University of La Plata (T06-01-2022).

### 4.4. Serum Parameters

At the end of the 21-day treatment period, the animals were fasted for 12 h and then euthanized by decapitation, and blood samples were collected. Glucose levels were immediately measured using test strips (Accu-Chek Performa Nano System, Roche Diagnostics, Mannheim, Germany). From the remaining blood, serum was obtained to determine the lipid profile, measuring triglycerides (TGs), total cholesterol, and HDL-cholesterol levels using enzymatic methods (TG Color GPO/PAP AA kit; Wiener Lab, Rosario, Argentina and Colestat Enz AA kit, Wiener Lab, Argentina, respectively). Non-HDL-cholesterol levels were calculated by the difference between total and HDL-cholesterol. The insulin resistance index (IRX) was determined by the TG/HDL- cholesterol ratio [41].

### 4.5. Intraperitoneal Glucose Tolerance Test (IGTT)

Animals from the 3 experimental groups were fasted for 12 h before intraperitoneal administration of 200 µL of a freshly prepared glucose solution (1.5 g/kg of body weight in saline buffer). Blood samples were collected under anesthesia from the retro-orbital plexus at 0, 15, 30, 60, 90, and 120 min after glucose challenge. Glucose concentration was measured as described in the previous section. Data are shown as the area under the curve (AUC) in mg/dL/120 min.

### 4.6. Pancreatic Islet Isolation

Immediately after euthanasia, the pancreases from all experimental animals were infiltrated with Krebs-Ringer bicarbonate (KRB) buffer (118 mM NaCl, 25.96 mM NaHCO_3_, 4.74 mM KCl, 2.24 mM CaCl_2_, 1.19 mM MgSO_4_, 0.91 mM KH_2_PO_4_; pH 7.4, previously gassed with a 5/95% CO_2_/O_2_ atmosphere) and fat was carefully removed for further pancreas digestion. Pancreatic islets were isolated with 0.02 mg/gr tissue of Collagenase-P (Roche, Mannheim, Ger many) at 37 °C with vigorous shaking until exocrine tissue was digested and islets were completely released.

For gene expression analysis by qPCR and Western blot, the isolated islets were previously washed twice with PBS buffer containing protease inhibitors (100 mM Benzamidine, 100 mM PMSF, and 4 mg Trasylol).

#### 4.6.1. Glucose-Induced Insulin Secretion

Five islets from each group were incubated for 60 min at 37 °C in 600 µL of gassed KRB buffer pH 7.4, containing 1% (*w/v*) BSA and 3.3 or 16.7 mM glucose [9]. After the incubation period, the medium was collected and stored at −20 °C for insulin determination by ELISA (Insulin mouse and rat EIA Kit, Cayman Chemical, Michigan, MI, USA). The results were expressed as ng of insulin/islet/hour. Ten tubes from each experimental condition were analyzed for each glucose concentration, 3.3 mM and 16.7 mM. Three independent experiments were conducted.

#### 4.6.2. Total RNA Isolation

Total RNA extraction from the isolated islets of the three experimental groups was performed using Trizol reagent (Gibco-BRL, Rockville, MD, USA). The 260/280 nm absorbance ratio was calculated to check for possible protein contamination and the integrity and purity of the extracted RNA were assessed by 1% agarose-formaldehyde gel electrophoresis. cDNA was synthesized using iScript reverse transcriptase from 1 µg of total RNA.

#### 4.6.3. RT-qPCR

For real-time PCR, we employed the iCycler 5 (Bio-Rad) and FastStart SYBR Green Master (Roche). The cycling conditions were as follows: 1 cycle of 1 min at 95 °C (DNA denaturation), and 40 cycles of 30 s at 95 °C, 30 s at 60 °C, and 30 s at 72 °C, followed by a melting curve analysis from 55 °C to 90 °C. Each PCR amplification was performed in triplicate. Primers were designed using PrimerSelect and EditSeq software 7.0.1, both from Lasergene. All amplicons included fragments between 100 and 200 bp size ranges. Data were expressed as relative gene expression normalized to the β-actin housekeeping gene (since it is expressed at similar levels in all cell types) and calculated using the ΔΔCT method. The oligonucleotide primers (Invitrogen, Buenos Aires, Argentina) used are listed in Table 3.

#### 4.6.4. Western Blot

Pancreatic islets isolated from all the experimental groups were homogenized in 80 mM Tris (pH 6.8), 5 mM EDTA, 5% sodium dodecyl sulphate (SDS), 10% glycerol, 5% dithiothreitol, and protease inhibitors (1 mM phenylmethylsulphonyl fluoride and 4 mg aprotinin). Subsequently, the samples were fractionated on SDS/PAGE (sodium dodecyl sulphate-polyacrylamide gel electrophoresis) and transferred to polyvinylidene difluoride transfer membrane (Amersham Hybond-P, GE Healthcare, Amershame, Bukinghamshire, UK) at constant 10 V for 30 min. Protein load was measured by the Bio-Rad protein assay by measuring absorbance at 595 nm. As a loading control, we used an antibody against GAPDH or β-actin, which were used as internal standards. This control was used to verify that all samples were loaded consistently on the gel and that differences in protein signals are not simply the result of variability in the amount of sample loaded and performed every run. For this reason, the results were expressed in arbitrary units (AUs) as the ratio between the protein of interest and β-actin or GAPDH band intensity. For each protein to be detected, membranes were horizontally cropped at the corresponding molecular weight. Precision Plus Protein Dual Color Standards (Bio-Rad) was used as a molecular weight marker. Cropped membranes were blocked with a 10% (*w/v*) non-fat milk solution for 2 h. Next, membranes were washed and incubated overnight at 4°C with specific primary antibodies [IR (CT-3-sc57342; 1:100), IRS-1 (H7-sc512070; 1:100), IRS-2 (BS-sc390761; 1:100), PI3K (D9-sc374534; 1:100), Bad (C7-sc8044; 1:100), Casp-8 ( H143-sc7890; 1:100) (sc: Santa Cruz, CA, USA), Casp-3 (C8487; 1:1000 Sigma, St. Louis, MA, USA) and 3-nitrotyrosine (621-44-3; 1:1000 Cayman, Michigan, MI, USA)]. The membranes were washed 3 times with Tween-Tris-buffered saline (T-TBS) and then incubated with the secondary antibody (anti-rabbit IgG-HRP or anti-mouse IgG-HRP) for 1 hour at room temperature. β-actin (A-1978; 1:10:000 Sigma, St. Louis, MA, USA) or GAPDH (C87727; 1:1000 Merck Millipore, Burlington, MA, USA). were used as an internal standard as appropriate. The proteins were visualized using an enhanced chemiluminescence detection system (ECL Prime, Amersham, GE Healthcare, UK). Finally, the membranes were scanned using the C-DiGit Blot scanner (LICOR), and the bands were quantified with Image Studio Digits 3.1 software. Periodically, to ensure that there are no non-specific signals generated by the binding of the secondary antibody to unrelated proteins, we performed a secondary antibody control where only the secondary antibody is applied without the primary antibody; a primary antibody control in which the primary antibody is applied in the absence of the protein of interest, confirming that the signal is not a product of nonspecific binding of the antibody; and a positive control that included a positive sample (in which the protein of interest is known to be present in high amounts) to ensure that the antibody can detect the protein of interest.

### 4.7. Statistical Analysis

The results are expressed as mean ± standard error of the mean (SEM). The normality of the variables was verified using the Shapiro–Wilk test. The differences were considered significant when *p* values were < 0.05. Statistical analysis was performed using GraphPad Prism 8.0.1 software, employing one-way ANOVA followed by Dunnett’s test for multiple comparisons. Bartlett’s test was used to assess the variance homogeneity.

## Figures and Tables

**Table 1:** Body weight and dietary parameters.

	C	F	I
Water intake (mL/rat/day)	36.06 ± 1.15	99.05 ± 4.45 *	78.97 ± 5.02 *
Food intake (g/rat/day)	23.94 ± 0.66	17.06 ± 0.70 *	15.78 ± 0.61 *
Calories consumed (kcal/rat/day)	68.06 ± 2.74	77.40 ± 2.68	73.59 ± 2.85
Body weight gain (g/day)	4.10 ± 0.23	4.31 ± 0.29	2.63 ± 0.25 #

Results are expressed as means ± SEM (*n* = 10 rats per group). * *p* < 0.05 vs C; # *p* < 0.05 vs. F.

**Table 2:** Serum parameters.

Plasma Parameters	C	F	I
Glucose (mg/dL)	129.1 ± 2.2	129.6 ± 1.8	131.8 ± 2.5
Triglyceride (mg/dL)	109.1 ± 7.9	264.3 ± 16.6 *	106.6 ± 13.5 #
HDL-cholesterol (mg/dL)	50.6 ± 2.5	41.3 ± 1.8 *	47.3 ± 2.3
IRX (TG/HDL)	2.3 ± 0.2	6.8 ± 0.5 *	2.3 ± 0.3 ^#^
Total cholesterol (mg/dL)	73.0 ± 2.7	74.7 ± 2.2	78.8 ± 3.5
Non-HDL-cholesterol (mg/dL)	22.4 ± 2.6	34.8 ± 2.1 *	28.8 ± 2.9
AUC (mg/dL/120 min)	19,272 ± 521.2	25,152 ± 1038 *	18,940 ± 1479 #

Values are expressed as means ± SEM (*n* = 10 rats per group). AUC, area under the glucose curve measured as mg/dL/120 min during IGTT. * *p* < 0.05 vs. C; # *p* < 0.05 vs. F.

**Table 3:** Specific primers used for real-time PCR analyses. FW: forward primer; and RV: reverse primer.

Gene	GeneBank^®^	Sequences
*IR*	NM_017071	FW 5′-ATATTGACCCGCCCCAGAGG-3′ RV 5′-TAGGTCCGGCGTTCATCAGA-3′
*IRS-1*	NM_012969	FW 5′-TGTGCCAAGCAACAAGAAAG-3′ RV 5′-ACGGTTTCAGAGCAGAGGAA-3′
*IRS-2*	NM_001168633.1	FW 5′-CTACCCACTGAGCCCAAGAG-3′ RV 5′-CCAGGGATGAAGCAGGACTA-3′
*PI3K*	NM_053481	FW 5′-TGCCCCATTTCATCCTTGTG-3′ RV 5′-GGTTGTTGTTGCCCCAGAC-3′
*IL- 1β*	NM_031512.2	FW 5′-CAAGGAGAGACAAGCAAGCAACGAC-3’ RV 5′-TCTTCTTTGGGTATTGTTTGGG-3’
*PAI-1*	NM_012620.1	FW: 5′-CCACGGTGAAGCAGGTGGACT-3′ RV: 5′-TGCTGGCCTCTAAGAAGGGG-3′
*TNF-α*	NM_0.126675.3	FW: 5′-GGCATGGATCTCAAAGACAACC-3′ RV: 5′-CAAATCGGCTGACGGTGTG-3′
*SOD-2*	NM_017051.2	FW: 5′-ACCGAGGAGAAGTACCACGA-3′ RV: 5′-TAGGGCTCAGGTTTGTCCAG-3′
*GPx1*	NM_030826.3	FW 5′-TGAGAAGGCTCACCCGCTCT-3′ RV 5′-GCACTGGAACACCGTCGTGA-3′
*GSR*	NM_053906.2	FW: 5′-TCTCCAAGCCCTTCATGAGTC-3′ RV: 5′-AGGCAAACACAATGCACATCC-3′
*Bad*	NM_022698.1	FW: 5′-CAGGCAGCAATAACAGTCA-3′ RV: 5′-CCCTCAAATTCATCGCTCAT-3′
*Bcl-2*	L14680.1	FW: 5′-CGGGAGAACAGGGTATGA-3′ RV: 5′-CAGGCTGGAAGGAGAAGAT-3′
*Casp-3*	NM_012922.2	FW 5′-AGCTGGACTGCGGTATTGAGA-3′ RV 5′-AACCATGACCCGTCCCTTGAA-3′
*Casp-8*	NM_022277.1	FW: 5′-TAAAAAGCAGCCCAGAGGAA-3′ RV: 5′-ATCAAGCAGGCTCGAGTTGT-3′
*mtTFA*	NM_031326.2	FWF: 5′-GAAAGCACAAATCAAGAGGAG-3′ RV: 5′-CTGCTTTTCATCATGAGACAG-3′
*PGC-1α*	NM_031347.1	FW: 5′-CAATGAATGCAGCGGTCTTA-3′ RV: 5′ ACGTCTTTGTGGCTTTTGCT-3′
*β-actin*	NM_031144.3	FW 5′-TGTCACCAACTGGGACGATA-3′ RV 5′-ACCCTCATAGATGGGCACAG-3′

## Data Availability

The original contributions presented in this study are included in the article. Further inquiries can be directed to the corresponding author.
